# The comparison of different acupuncture therapies for post stroke depression: A protocol for a Bayesian network meta-analysis

**DOI:** 10.1097/MD.0000000000023456

**Published:** 2020-12-24

**Authors:** Chang Rao, Wei Liu, Zefang Li, Xi Nan, Chunsheng Yin, Jipeng Yang, Yuzheng Du

**Affiliations:** First Teaching Hospital of Tianjin University of Traditional Chinese Medicine, National Clinical Research Center for Chinese Medicine Acupuncture and Moxibustion, Tianjin, China.

**Keywords:** acupuncture, network meta-analysis, post stroke depression, protocol

## Abstract

**Background:**

Depression is a common disease which occurs after stroke, affecting approximately one third of stroke survivors at any 1 time after stroke (compared with 5%–13% of adults without stroke), with a cumulative incidence of 55%. Acupuncture, which has a long history in China, is the generic name of different kinds of acupuncture therapies, including manual acupuncture (MA), electroacupuncture (EA), fire needle (FN), dry needling (DN), and so on. Clinical studies have shown that acupuncture has a good therapeutic effect on post stroke depression (PSD), but the evidence-based medicine of it is insufficient. The purpose of this study is to systematically evaluate the efficacy of different kinds of acupuncture therapies in the treatment of PSD, and to provide evidence-based basis for the clinical application of acupuncture in the treatment of PSD.

**Methods:**

A systematic search will be performed on English databases (PubMed, The Cochrane Library, Medline, Embase) and Chinese databases (China National Knowledge Infrastructure (CNKI), WanFang Data, VIP and Chinese biomedical databases). The retrieval time limit will be from the establishment of the database to August 2020. Two researchers will independently screen the literatures, extract data, and evaluate the quality of the included studies. Bayesian network analysis will be conducted by using STATA V.14.0 and ADDIS V.1.16.7.

**Results:**

In this study, the efficacy of different kinds of acupuncture therapies in the treatment of PSD will be evaluated by the degree of reduction in depression, total numbers of adverse events, quality of life indices, improvement of social and life functions and the expression of nerve cell factors.

**Conclusions:**

This study will provide reliable evidence-based evidence for the clinical application of acupuncture in PSD.

## 1 Introduction

### 1.1 Description of the condition

Stroke was the leading cause of death, disability-adjusted life-years (DALYs), and years of life lost (YLLs) in China according to the latest global burden of disease study.^[1]^ Depression could occur in approximately one third of stroke survivors at any time, which would lead to poorer functional outcomes, quality of life (QOL), and higher mortality.^[2–4]^ Although using selective serotonin uptake inhibitors (SSRIs) or serotonin and norepinephrine reuptake inhibitors (SNRIs) as first-line treatment for depressive disorder was supported by the Canadian network for mood and anxiety treatments (CANMAT),^[5]^ the American psychiatric association (APA)^[6]^ and the World federation of societies of biological psychiatry (WFSBP)^[7]^ guidelines, the etiopathogenesis and pathophysiology of depressive disorder is incomplete.^[8]^

Few guidelines exist regarding the assessment, treatment, and prevention of post stroke depression (PSD).^[9]^ Although antidepressant drugs may benefit people with persistent depressive symptoms after stroke, it should be used with caution, as little is known about the side effects.^[10]^ A large Cochrane systematic review^[11]^ including 63 trials with 9168 ischemic or hemorrhagic stroke survivors showed that there was not enough reliable evidence to support using SSRIs as the routine drugs to promote recovery after stroke, although it could reduce the average depression score(SMD 0.11 lower, 0.19 lower to 0.04 lower; 2 trials, 2861 participants; moderate-quality evidence). What is more, no clear consensus has been reached regarding to the timing of antidepressants or the discontinuation of adjunctive psychotropics in the treatment of depression.^[12]^ In the future, greater efforts are required to improve the diagnosis and management for PSD.

### 1.2 Description of the intervention

Acupuncture, which has a long history in China, is the generic name of different kinds of acupuncture therapies, including manual acupuncture (MA), electroacupuncture (EA), fire needle (FN), dry needling (DN), and so on. MA is most used as research object to study the therapeutic effect of acupuncture. Different kinds of acupuncture therapies may differ in their effectiveness, a narrative review^[13]^ suggested that there were clear dose-response relationships between menstrual pain outcomes and acupuncture “dose”, including type of acupuncture therapy, needle location, number of needles used, and frequency of treatment. However, little research has been conducted to directly examine effects on clinical outcomes when these parameters of acupuncture are changed.^[14]^

In recent years, improving depressive symptoms by acupuncture therapies have received more and more attention. A Cochrane review^[15]^ which included 63 trials with 7060 participants examined the effectiveness of acupuncture for depression, and found that severity of depression was significantly reduced during treatment for acupuncture compared with no treatment/wait list/treatment as usual. However, the possible benefit of acupuncture provided alone or in conjunction with pharmaceutical medication such as SSRIs compared with pharmaceutical monotherapy is unclear, mostly due to the very low quality of available evidence. In the other hand, there were less reviews focused on the effectiveness of acupuncture in treating PSD. An overview of meta-analysis^[16]^ result of acupuncture in the treatment of PSD showed that acupuncture was potentially effective and safe monotherapy for post stroke depression, however, there were insufficient researches in this area. What is more, most of the meta-analysis did not attach importance to the comparison of different acupuncture therapies.^[17,18]^

### 1.3 Objectives

Objectives of this systematic review and network meta-analysis are to:

1. compare and rank all acupuncture therapies in terms of efficacy in the treatment of PSD; and
2. explore the relationship between acupuncture “dose” and the depression improving outcome.

## 2 Methods

Because it is a network meta-analysis, there is no need for ethics.

### 2.1 Study registration

This network meta-analysis has been registered on INPLASY network (Registration number: INPLASY2020100104).

### 2.2 Eligibility criteria

#### 2.2.1 Types of studies

Randomized controlled trials (RCTs) in English or Chinese will be included. There are no restrictions on publication date. Animal studies or studies with incomplete data will be excluded.

#### 2.2.2 Participants

We will include RCTs that involved patients who suffered post stroke depression. The diagnosis of stroke should base on computer tomography (CT), magnetic resonance imaging (MRI), or clinical criteria. Meanwhile, depression were diagnosed according to the International Classification of Diseases, Tenth Edition (ICD-10),^[19]^ the Diagnosis and Statistical Manual of Mental Disorders (DSM),^[19]^ Chinese Classification of Mental Disorders (CCMD),^[20]^ or the 17-item Hamilton Rating Scale for Depression (HAMD-17).^[21]^

#### 2.2.3 Types of interventions

The RCTs with experiment group(EG) and control group(CG) meeting one of the following conditions will be included:

1. the intervention of EG is the therapy based on acupuncture(including acupuncture alone, or the acupuncture plus SSRIs), the intervention of CG is SSRIs;
2. using acupuncture plus placebo as the intervention of EG, and SSRIs plus sham acupuncture as the or the intervention of CG
3. using different kinds of acupuncture therapies as the intervention of CG and EG respectively.

Our selection of acupuncture therapy for analysis will include MA, EA, FN, DN, laser needle(LN), or the combination of different kinds of acupuncture therapies, regardless of acupoint selection or acupuncture manipulation. According to our preliminary search result in the database, we found that there were few trials focused on the comparison between different kinds of acupuncture therapies, whereas more trials were focused on the comparison between acupuncture and western medicine. Therefore, we chose the SSRIs which had become the first-line antidepressant medication class over the last quarter of a century^[22]^ as the common comparison object, in order to achieve the comparison between different kinds of acupuncture therapies.

#### 2.2.4 Types of outcome measures

Primary outcomes

1. Degree of reduction in depression, measured by self-rating scales such as Beck Depression Inventory (BDI) or clinician-rated rating scale such as Hamilton Depression Rating Scale (HAMD);
2. Total numbers of adverse events.

Secondary outcomes

1. Quality of life indices such as the World Health Organization Quality of Life (WHOQOL);
2. Improvement of social and life functions such as Activity of Daily Living Scale (ADL);
3. The expression of nerve cell factors, such as serotonin (5-HT), IL-23.

There are several scales used for assessment of depression, for example HAMA, MADRS, SDS, BDI, if 2 or more scales are used in a research, we apply the following hierarchy:

1. HAMD,
2. MADRS;
3. CGI-S;
4. BDI;
5. PHQ and
6. All other depression scales.

If researchers used more than 1 quality of life measure, we apply the following hierarchy of scales:

1. World Health Organization Quality of Life,
2. WHO Quality of Life-BREF (WHOQOL-BREF),
3. Short Form Health Survey and
4. Any other quality of life measures used.

#### 2.2.5 Data source

The following databases will be searched from inception to July 2020: PubMed, The Cochrane Library, Medline, Embase, China National Knowledge Infrastructure (CNKI), WanFang Data, VIP and Chinese Biomedical Databases. The combination of free words and medical subject headings, including “depression, depressive disorder, acupuncture therapy, acupuncture, electroacupuncture, dry needle, stroke, etc”, will be used as the retrieval mode. The search strategy for PubMed is shown in Table 1.

**Table 1:** Search strategy in PubMed database.

Number	Search terms
#1	Stroke [Title/Abstract]
#2	Apoplexy [Title/Abstract]
#3	Cerebral Hemorrhage [Title/Abstract]
#4	Cerebrum Hemorrhage [Title/A bstract]
#5	Cerebral Infarction [Title/Abstract]
#6	Cerebral Infarct [Title/Abstract]
#7	#1 OR #2 OR #3 OR #4 OR #5 OR #6
#8	Acupuncture [Title/Abstract]
#9	Electroacupuncture [Title/Abstract]
#10	Laser Needle [Title/Abstract]
#11	Fire Needle [Title/Abstract]
#12	Dry Needling [Title/A bstract]
#13	#8 OR #9 OR #10 OR #11 OR #12
#14	Depression [Title/Abstract]
#15	Depressive [Title/Abstract]
#16	#14 OR #15
#17	#7 AND #13 AND #16

#### 2.2.6 Study selection

NoteExpress V.3.2 will be used to manage studies. First, duplicate literature will be removed through electronic/manual-based steps in NE. Second, 2 reviewers will independently screen the study article titles and abstracts and select the studies which meet the eligibility criteria. If there are disagreements, the third reviewer should be consulted. Two reviewers independently extracted the general information of the included studies. Extracted data include authors, year of publication, sample size, source of diagnosis, the observation period, interventions and test results of all groups. If there is any necessary data missing, corresponding authors will be contacted and asked to provide relevant details. Some studies will be excluded if the full text or data are unable to get access and the reasons for exclusion will be reported in detail in these cases.

**Figure 1:**
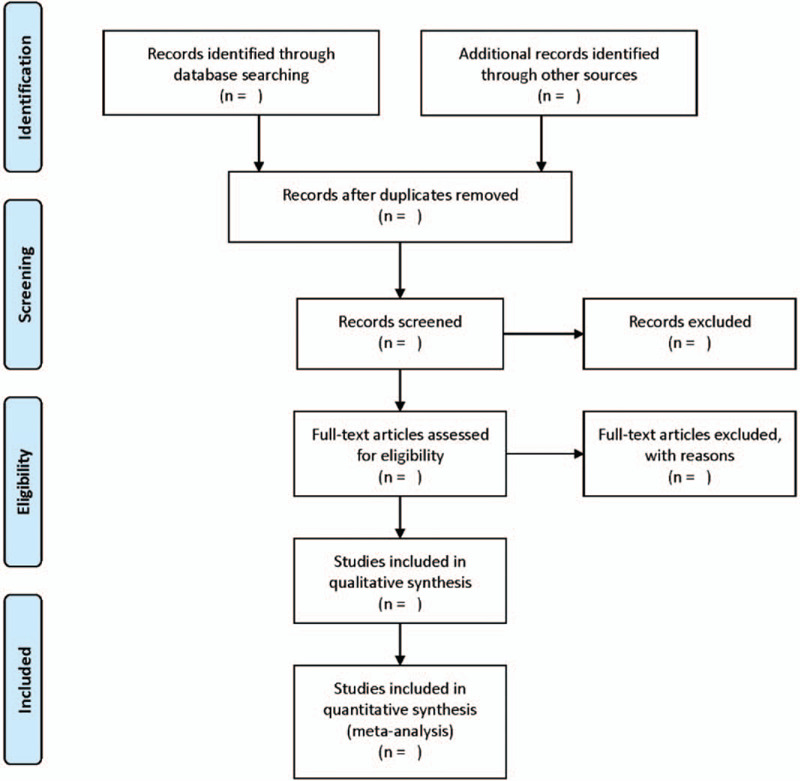
Flow diagram. This picture reflects the steps of research selection, and explains the process of literature screening in detail.

The entire process will be presented by the PRISMA flow chart (http://www.prismastatement.org). The literature screening process is shown in Figure 1.

#### 2.2.7 Risk of bias

According to Cochrane Handbook for Systematic Reviews of Interventions version 6(https://training.cochrane.org/handbook/current/chapter-08), the methodological quality and the risk of bias of the included studies by means of the risk of bias 2.0(ROB 2.0) tool. One researcher assessed the risk of bias of included studies by using ROB 2.0 and the other researcher confirmed the judgment. Any differences will be resolved through discussions with the third researcher. The following items were categorized as having high, low or unclear risk of bias: random sequence generation, allocation concealment, blinding of participants and personnel, blinding of outcome assessment, incomplete outcome data, selective reporting, and other biases which focusing on baseline imbalance.

#### 2.2.8 Network meta-analysis

Bayesian network analysis will be conducted to compare the effects of different acupuncture therapies. STATA V.14.0 and ADDIS V.1.16.7 will be used to perform the network meta-analysis. We will use the *I*^2^ statistic will to assess the heterogeneity. If the *I*^2^ value is below 50%, the fixed effect model will be used. Otherwise, sensitivity analysis and subgroup analysis will be conducted to explore the main sources of heterogeneity, after which, the random effect model will be used if the *I*^2^ is still equal or greater than 50%. Both types of effect sizes will be presented with 95% CIs, and values of *P* < .05 will be regarded as statistically significant. Continuous outcomes will be calculated as mean differences (MDs) or standardized mean differences (SMDs), meanwhile, binary outcomes will be calculated as odds ratios (ORs).

The results of node splitting will be used to evaluate the inconsistency between direct and indirect evidence. If there is no relevant inconsistency in the evidence, a consistency model can be used to draw conclusions about the relative effect of the included treatments. Convergence is assessed using the Brooks-Gelman-Rubin method, which could compare the within-chain and between-chain variance to calculate the Potential Scale Reduction Factor (PSRF).^[23]^ The simulation iteration number will be set to 50,000 and the tuning iteration number will be initially set to be 20,000. Meanwhile, the Bayesian approach will also be used to estimate the rank probabilities. A rank probability plot will be given to show the priority of different acupuncture therapies.

Evidence supporting the relationships of the included studies will be determined by analysis of a network plot, and resultant figures and network meta-analysis graphs will be presented.

#### 2.2.9 Quality of evidence

The Grades of Recommendation, Assessment, Development, and Evaluation (GRADE) approach will be used to assess the quality of evidence for main outcomes. Two reviewers will do this independently through GRADEpro GDT (https://gradepro.org/). GRADE approach provides guidance for rating quality of evidence and grading strength of recommendations in health care. It has important implications for those summarizing evidence for systematic reviews.^[24]^ It assessed a body of evidence by referring to the concepts of the GRADE system, and determined and recorded the quality of a body of evidence for each clinical question, there are 4 quality levels: high, moderate, low, and very low.^[25]^

## 3 Discussion

PSD affects the prognosis of patients. Some evidences^[26,27]^ suggest that the severity of disability after stroke correlates with the degree of depression. Clinical studies^[28]^ have shown that at the similar stroke level, PSD patients showed a significant decline in activity of daily living and a more severe degree of handicap than non-PSD patients. PSD may also aggravate cognitive impairment in stroke patients.^[29]^

In China, acupuncture, an important part of TCM, is commonly used in hospitals as an important role in clinical treatment.^[30]^ On the one hand, acupuncture can assist in eliminating bad emotions by significantly improving the functional communication and language function,^[31]^ cognitive^[32]^ and limb movement function^[33]^ of stroke patients. On the other hand, acupuncture can modulate glutamate receptor and EAAT expression,^[34]^ down-regulated the levels of NF-κB protein, iNOS and NO,^[35]^ so as to achieve the purpose of relieving PSD.

As far as we know, NMA has not been used to compare the effectiveness of different acupuncture therapies for PSD in recent years. Furthermore, we will use the GRADE approach to assess the quality of evidence for main outcomes. But the limitation of language will lead to some bias. Besides, the lack of literature on acupuncture treatment of PSD which are large sample size and high quality may affect the authenticity of this study.

## Author contributions

**Data curation:** Chang Rao, Wei Liu.

**Funding acquisition:** Yuzheng Du.

**Investigation:** Jipeng Yang.

**Resources:** Zefang Li, Xi Nan.

**Software:** Zefang Li, Xi Nan, Chunsheng Yin.

**Supervision:** Yuzheng Du.

**Writing – original draft:** Chang Rao, Wei Liu.

**Writing – review & editing:** Chang Rao, Wei Liu.
